# Disease correlates of rim lesions on quantitative susceptibility mapping in multiple sclerosis

**DOI:** 10.1038/s41598-022-08477-6

**Published:** 2022-03-15

**Authors:** Melanie Marcille, Sandra Hurtado Rúa, Charles Tyshkov, Abhishek Jaywant, Joseph Comunale, Ulrike W. Kaunzner, Nancy Nealon, Jai S. Perumal, Lily Zexter, Nicole Zinger, Olivia Bruvik, Yi Wang, Elizabeth Sweeney, Amy Kuceyeski, Thanh D. Nguyen, Susan A. Gauthier

**Affiliations:** 1 Department of Neurology, Weill Cornell Medicine, New York, NY USA; 2 Department of Mathematics and Statistics, Cleveland State University, Cleveland, OH USA; 3 Department of Pediatrics, Weill Cornell Medicine, New York, NY USA; 4 Department of Psychiatry and Rehabilitation Medicine, Weill Cornell Medicine, New York, NY USA; 5 Department of Radiology, Weil Cornell Medicine, New York, NY USA; 6 Feil Family Brain and Mind Institute, Weill Cornell Medicine, New York, NY USA; 7 Department of Population Health Sciences, Weill Cornell Medicine, New York, NY USA

**Keywords:** Multiple sclerosis, Magnetic resonance imaging

## Abstract

Quantitative susceptibility mapping (QSM), an imaging technique sensitive to brain iron, has been used to detect paramagnetic rims of iron-laden active microglia and macrophages in a subset of multiple sclerosis (MS) lesions, known as rim+ lesions, that are consistent with chronic active lesions. Because of the potential impact of rim+ lesions on disease progression and tissue damage, investigating their influence on disability and neurodegeneration is critical to establish the impact of these lesions on the disease course. This study aimed to explore the relationship between chronic active rim+ lesions, identified as having a hyperintense rim on QSM, and both clinical disability and imaging measures of neurodegeneration in patients with MS. The patient cohort was composed of 159 relapsing–remitting multiple sclerosis patients. The Expanded Disability Status Scale (EDSS) and *Brief International Cognitive Assessment for Multiple Sclerosis*, which includes both the Symbol Digit Modalities Test and California Verbal Learning Test-II, were used to assess clinical disability. Cortical thickness and thalamic volume were evaluated as imaging measures of neurodegeneration. A total of 4469 MS lesions were identified, of which 171 QSM rim+ (3.8%) lesions were identified among 57 patients (35.8%). In a multivariate regression model, as the overall total lesion burden increased, patients with at least one rim+ lesion on QSM performed worse on both physical disability and cognitive assessments, specifically the Symbol Digit Modalities Test (p = 0.010), California Verbal Learning Test-II (p = 0.030), and EDSS (p = 0.001). In a separate univariate regression model, controlling for age (p < 0.001) and having at least one rim+ lesion was related to more cortical thinning (p = 0.03) in younger patients (< 45 years). Lower thalamic volume was associated with older patients (p = 0.038) and larger total lesion burden (p < 0.001); however, the association did not remain significant with rim+ lesions (p = 0.10). Our findings demonstrate a novel observation that chronic active lesions, as identified on QSM, modify the impact of lesion burden on clinical disability in MS patients. These results support further exploration of rim+ lesions for therapeutic targeting in MS to reduce disability and subsequent neurodegeneration.

## Introduction

Innate immunity plays a pivotal role in the pathophysiology of multiple sclerosis (MS), and important cell types involved in this process are CNS resident monocytes (microglia) and blood-derived macrophages^1^. Chronic CNS inflammation in the MS lesion is maintained, in part, with pro-inflammatory microglia and macrophages at the rim of chronic active or slowly expanding MS lesions, which are the site of ongoing demyelination and axonal damage^2^. The continued expansion of chronic active lesions has been postulated to play an essential role in the pathogenesis of disease progression in MS^3^.

A significant proportion of microglia and macrophages found at the rim of chronic active MS lesions contain iron, distinguishing them from chronic inactive lesions where iron is essentially absent^4–7^. Gradient echo (GRE) MRI sequences are sensitive to iron^8^ and have been used to explore iron dynamics in the brain^9^. Detection of a paramagnetic rim in a subset of chronic lesions has generated significant interest, and numerous in vivo and histopathological validation studies have confirmed that the rim is representative of iron-laden inflammatory cells^4–6,10^. These studies have provided further evidence that chronic active lesions can occur throughout the disease course, including within patients with preclinical disease^11,12^, and can be associated with clinical disability^13^. Quantitative susceptibility mapping (QSM)^14^ is an effective post-processing technique for GRE data that directly maps the various sources of susceptibility by solving the field-to-source inversion problem^15^ and providing quantification and localization of brain iron^16–19^. Lesions with a hyperintense rim (rim+) on QSM have increased inflammation on PK11195-PET, with histopathological and in vitro studies confirming a rim of inflammatory cells and more tissue damage on myelin water fraction imaging^15,20,21^. The quantitative nature of QSM provides a unique opportunity to capture time-dependent changes of rim lesions, as their susceptibility remains high even after years of initial detection and slowly decays over time^10,22–24^.

The association between chronic active MS lesions and disability has been studied utilizing various GRE imaging methods, which have ultimately suggested a negative impact of these lesions on disease course^13,25,26^. The goal of this study was to expand upon earlier studies and assess the association of rim+ lesions, as detected on QSM, with clinical disability, global measures of structural tissue damage, and their interactions in a large cohort of remitting (RR) MS patients. To assess the impact on clinical disability, we explored the relationship between chronic rim+ lesions on both cognitive function and standard measures of neurological disability. Gray matter atrophy, as an MRI measure of neurodegeneration, occurs early and has the strongest association with long-term clinical disability^27–29^; thus, in a secondary analysis, we further explored the impact of rim+ lesions on measures of cortical and thalamic integrity.

## Methods

### Patient cohort

This was a cross-sectional study of 159 patients with RRMS meeting the following inclusion criteria: (1) 2010 McDonald criteria^30^, (2) age ≥ 18 years and (3) already participants within our research repository [willing (and reconsented) to have an annual cognitive evaluation at the time of their annual MRI for a total of 5 years]. The current study is the baseline analysis of a longitudinal study. Exclusion criteria were the following: (1) clinically isolated syndrome or progressive forms of MS, (2) technical limitations of MRI, or (3) standard of care MRI scan not completed within 45 days of the BICAMS assessment (study flow chart included in the Supplementary File). For this cohort, cognitive and neurological assessments were completed within an average of 14 ± 21 days of their annual MRI scan. Progressive MS phenotypes were excluded from this study in an attempt to provide novel insight into the prevalence of chronic active lesions at earlier stages of the disease. Cognitive function was evaluated utilizing *The Brief International Cognitive Assessment for Multiple Sclerosis* (BICAMS), which is composed of three assessments: Symbol Digit Modalities Test (SDMT), California Verbal Learning Test-II (CVLT-II) Immediate Recall (Total of Trials 1–5), and Brief Visuospatial Memory Test-Revised (BVMT-R) Immediate Recall (Total of Trials 1–3)^31^. SDMT assesses processing speed, CVLT-II measures verbal learning and short-term memory, and BVMT-R examines visuospatial learning and short-term memory. A lower score for each cognitive assessment is associated with a lower cognitive performance. Expanded Disability Status Score (EDSS) was utilized to measure neurological dysfunction, with a higher EDSS score indicating increased disability. The study was approved by an ethical standards committee on human experimentation at Weill Cornell Medicine, and written informed consent, which provides permission to store and analyze clinical and MRI data, was obtained from all patients according to the Declaration of Helsinki.

The following clinical data were also collected for all patients: gender, age, disease duration from initial symptom, duration on current disease-modifying treatments (DMT), and disease subtype. Treatments were groups as the following: (a) intravenous (IV): ocrelizumab, alemtuzumab, natalizumab, rituximab, or IV immunoglobulin; (b) oral: fingolimod, dimethyl fumarate, teriflunomide, and (c) injection therapy: glatiramer acetate and beta-interferon.

### MRI protocol and image processing

Imaging was performed on a 3T Magnetom Skyra scanner (Siemens Medical Solutions USA, Malvern, PA) using a product twenty-channel head/neck coil. The MRI protocol consisted of sagittal 3D T1-weighted (T1w) sequence for anatomical structure, 2D T2-weighted (T2w) fast spin echo, and 3D T2w fluid attenuated inversion recovery (FLAIR) sequences for lesion detection, gadolinium-enhanced 3D T1w sequence for acute lesion identification, and axial 3D multi-echo GRE sequence for QSM. Detailed description of the imaging protocol: (1) 3D sagittal T1w MPRAGE: Repetition Time (TR)/Echo Time (TE)/Inversion Time (TI) = 2300/2.3/900 ms, flip angle (FA) = 8°, GRAPPA parallel imaging factor (R) = 2, voxel size = 1.0 × 1.0 × 1.0 mm^3^; (2) 2D axial T2-weighted (T2w) turbo spin echo: TR/TE = 5840/93 ms, FA = 90°, turbo factor = 18, R = 2, number of signal averages (NSA) = 2, voxel size = 0.5 × 0.5 × 3 mm^3^; (3) 3D sagittal fat-saturated T2w fluid attenuated inversion recovery (FLAIR) SPACE: TR/TE/TI = 8500/391/2500 ms, FA = 90°, turbo factor = 278, R = 4, voxel size = 1.0 × 1.0 × 1.0 mm^3^; (4) *axial 3D multi-echo GRE sequence for QSM:* axial field of view (FOV) = 24 cm, TR/TE1/ΔTE = 48.0/6.3/4.1 ms, number of TEs = 10, FA = 15°, R = 2, voxel size = 0.75 × 0.93 × 3 mm^3^, scan time = 4.2 min. QSM was reconstructed from complex GRE images using a fully automated Morphology Enabled Dipole Inversion algorithm zero-referenced to the ventricular cerebrospinal fluid (MEDI + 0)^32^.

### Brain tissue segmentation

FreeSurfer^33^ was utilized for the segmentation of white matter (WM) and gray matter (GM) on T1w images. The GM segmentation masks were checked, and lesion in-painting was completed to prevent misclassification due to T1-hypointensities associated with lesions^34^.

Cortical thickness was computed using FreeSurfer, available through the website (http://www.surfer.nmr.mgh.harvard.edu/). All the surface reconstruction results were generated using the fully automated processing pipeline. For each point on the gray/white surface, the shortest distance to the pial surface is first computed. Next, for each point on the pial surface, the shortest distance to the gray/white surface is found, and the cortical thickness at that location is set to the average of these two values^35^.

FreeSurfer software was used to obtain thalamic volumes for all subjects. Thalamic segmentations are based on the assignment of neuroanatomical labels to each voxel in an MR image based on the probabilistic information automatically estimated from a manually labeled training set. The methods of the automated volumetric approach have been described in detail previously^36^. Since structures may scale with the general head size, an automated method was used to normalize thalamic volume to the overall intracranial volume^37^.

### Lesion segmentation and QSM rim identification

MS lesions were segmented on FLAIR images by an automated lesion growth algorithm as implemented in the LST toolbox version 3.0.0 (www.statisticalmodelling.de/lst.html), followed by manual editing and creation of individual lesion labels on the FLAIR sequence. Defining individual lesions can be difficult; therefore, T1 and T2 images were referenced, as needed, during the editing process to best define individual lesion boundaries. The automated lesion labels were extended or erased to match the original lesion signal. The edited lesion mask on FLAIR was then co-registered to QSM.

**Figure 1:**
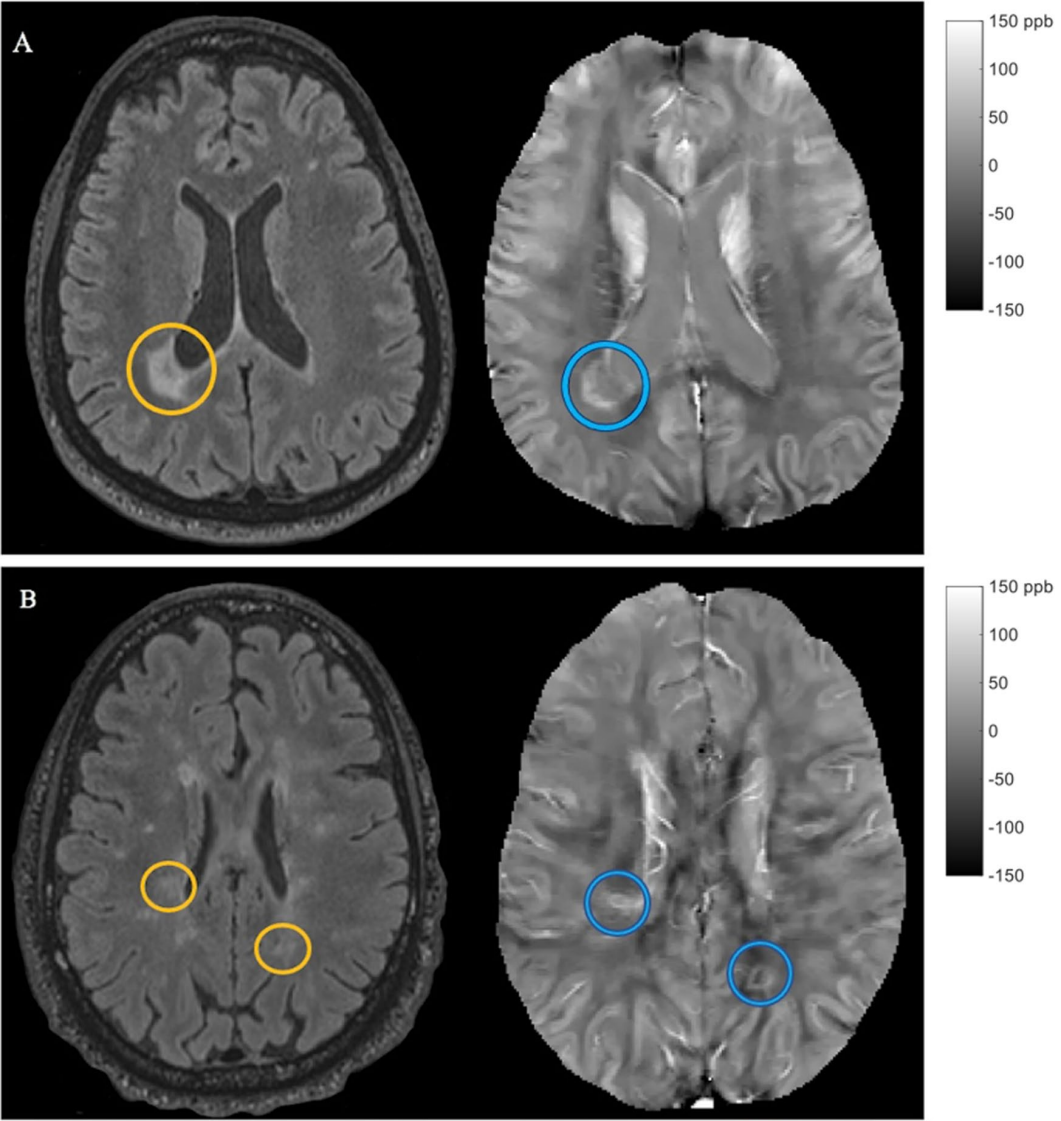
Examples of QSM rim+ lesions. Panels (**A**) and (**B**) represent slices of imaging for two separate patients. Within each panel, FLAIR is located on the left, and the corresponding slice of QSM is located on the right. In both panels (**A**) and (**B**), the lesion on FLAIR is located within an orange circle, while the corresponding lesion on QSM is located within a blue circle. These examples represent lesions with a complete rim on QSM.

Chronic active lesions with paramagnetic rims were identified by two trained independent raters on QSM, one of whom is a neuroradiologist, and another trained independent rater then reviewed and resolved the discrepant lesions. Each reviewer was trained on the identification of rim lesions on QSM by an imaging data specialist (SG) with eight years of experience with QSM lesion analysis. As part of their training, the reviewers identified sample rim lesions and then compared their identifications to each other and a specialist in order to ensure consistency and accuracy. As represented in Fig. 1, a lesion on FLAIR imaging was labeled as rim+ based on the presence of a hyperintense rim, relative to the lesion core, on QSM imaging. Lesions having a partial or complete rim were considered to be rim+.

Within the current study, the two initial independent raters only disagreed on the classification of 159 lesions yielding an initial agreement of 95.9% (Bangdiwala's B-statistics = 95.49%). The third reviewer resolved the differences between the two initial raters, yielding Cohen’s Kappa coefficients of 70.6% and 85.8% with raters one and two, respectively^38^. The Fleiss coefficient for all three raters was equal to 71%.

### Statistical analysis

Two-sided p-values were computed using the Welch two-sample t-test or Mann–Whitney test for continuous symmetric variables to compare patients with and without rim lesions (Table 1). A chi-square test was used to compare categorical variables between patient groups. A multivariate regression model was implemented to test the association between clinical scores (SDMT, CVLT-II, BVMT-R, and EDSS) as a response vector and the number of rim+ lesions per patient while adjusting for total FLAIR lesion volume (TLV). In addition, the model accounts for other patient-level covariates such as gender, age, disease duration, and duration of current treatment, as well as covariate interactions (see online Supplementary File for a full description of the model). The combined assessment of cognitive function and EDSS was implemented to evaluate disability more comprehensively. The model is defined as multivariate^39^ because it measures the association between four response variables (SDMT, CVLT-II, BVMT-R, and EDSS) and covariates of interest. A multivariate regression model allows us to quantify the relationship between all four disability scores as response variables and multiple patient-level covariates in one framework, thus providing more powerful tests of significance as compared to running four independent univariate models^40^. Model assumptions were checked, and variable transformations were analyzed using descriptive plots and summary tables to improve linearity and normality. The only variable transformation included for modeling purposes was $log(TLV.flair)$ and $log\left(EDSS+0.1\right)$; however, model-based mean estimates (SDMT, CVLT-II, BVMT-R, and EDSS) were reported using their original scale. For cognitive measures, we report descriptively the percentage of our sample who fell in the clinically impaired range based on published normative data^41–43^; however, we used raw scores rather than age-normed scores for statistical analyses because age was already included in our models as a covariate.

**Table 1:** Clinical and MRI characteristics of all patients. Abbreviations include expanded disability status score (EDSS), symbol digit modality test (SDMT), California verbal learning test-II (CVLT-II), and brief visuospatial memory test-revised (BVMT-R). Thalamic volume is unitless (normalized to head size). Significant values are in bold.

Characteristics	Patients with zero rim+ lesions (n = 102)	Patients with at least one rim + lesion (n = 57)	p-value
Female, n (%)	76 (74.51)	39 (68.42)	0.523
Disease duration (years), mean (SD)	11.02 (8.05)	10.24 (7.37)	0.528
Age (years), mean (SD)	41.96 (17.58)	42.54 (10.14)	0.731
Current treatment duration, mean (SD)	3.80 (4.28)	2.88 (3.32)	0.131
**Cognitive score, mean (SD)**			
SDMT	58.31 (11.14)	52.70 (11.78)	**0.004**
CVLT-II	54.34 (8.42)	50.86 (11.95)	**0.028**
BVMT-R	25.27 (5.93)	22.88 (7.57)	**0.032**
**Cognitive impairment, n (%)**			
SDMT	15 (14.71)	20 (35.09)	**0.005**
CVLT-II	6 (5.88)	10 (17.54)	**0.038**
BVMT-R	21 (20.59)	20 (35.09)	0.069
EDSS, median (IQR)	0.5 (2)	1.50 (2.5)	**0.025**
**MRI measures**			
Cortical thickness (mm), mean (SD)	2.53 (0.09)	2.48 (0.09)	**0.002**
Thalamic volume, mean (SD)	0.0093 (0.0009)	0.0089 (0.0008)	**0.0051**
No. lesions on FLAIR, median (IQR)	13 (20)	34 (31)	**< 0.001**
Lesion volume on FLAIR (mm^3^), mean (SD)	3127.72 (4619.32)	10,296.04 (10,412.46)	**< 0.001**

Two univariate regression models were also implemented to test the association between both cortical thickness and thalamic volume and the number of rim+ lesions at the patient level, while controlling for gender, age, disease duration, duration of current treatment, and TLV.

All final models were selected using a stepwise backward procedure with a 0.10 significance level. Inferences are based on a 5% significant level. Furthermore, model-based means were computed along with their 95% confidence intervals. Statistical analysis was performed using R: A language and environment for statistical computing R Core Team (2020).

## Results

### Patient cohort

This was a cross-sectional study composed of 159 patients with RRMS. The cohort included 115 females and 44 males aged (mean ± SD) 42.17 ± 10.25 years (years), current treatment duration of 3.47 ± 3.97 years, disease duration of 10.74 ± 7.51 years, and median Expanded Disability Status Scale of 1.0 (IQR = 2). Regarding treatment type at the time of cognitive testing, the majority (37.1%) were on IV, 34.0% on oral, and 15.7% were on injectable therapies. 13.2% percent of the patients were untreated. The proportion of cognitive impairment found among the entire patient cohort was relatively low and observed to be the following: 22.01% on SDMT, 10.06% on CVLT-II, and 25.79% on BVMT-R.

### QSM rim+ lesions

The median number of FLAIR lesions identified was 20 (IQR = 33.5), and only 5.7% of patients had evidence of Gd enhancing lesions (20 lesions). We identified 171 (3.8%) lesions that were classified as rim+ (Fig. 1), and none of the rim+ lesions were enhancing with Gd. The B-test agreement between the two main raters was high (B-statistics = 95.49%). The number of QSM rim+ lesions per patient ranged from zero to a maximum of 17 (Median = 0, IQR = 1). One hundred and two patients (64.2%) did not have any rim+ lesions. Of the fifty-seven patients (35.8%) with at least one rim+ lesion, the median number of rim+ lesions per patient was 2 (IQR = 3). Table 1 presents the clinical and MRI characteristics among patients without rim+ lesions as compared to those with at least one rim+ lesion.

### Association of QSM rim lesions and disability

**Figure 2:**
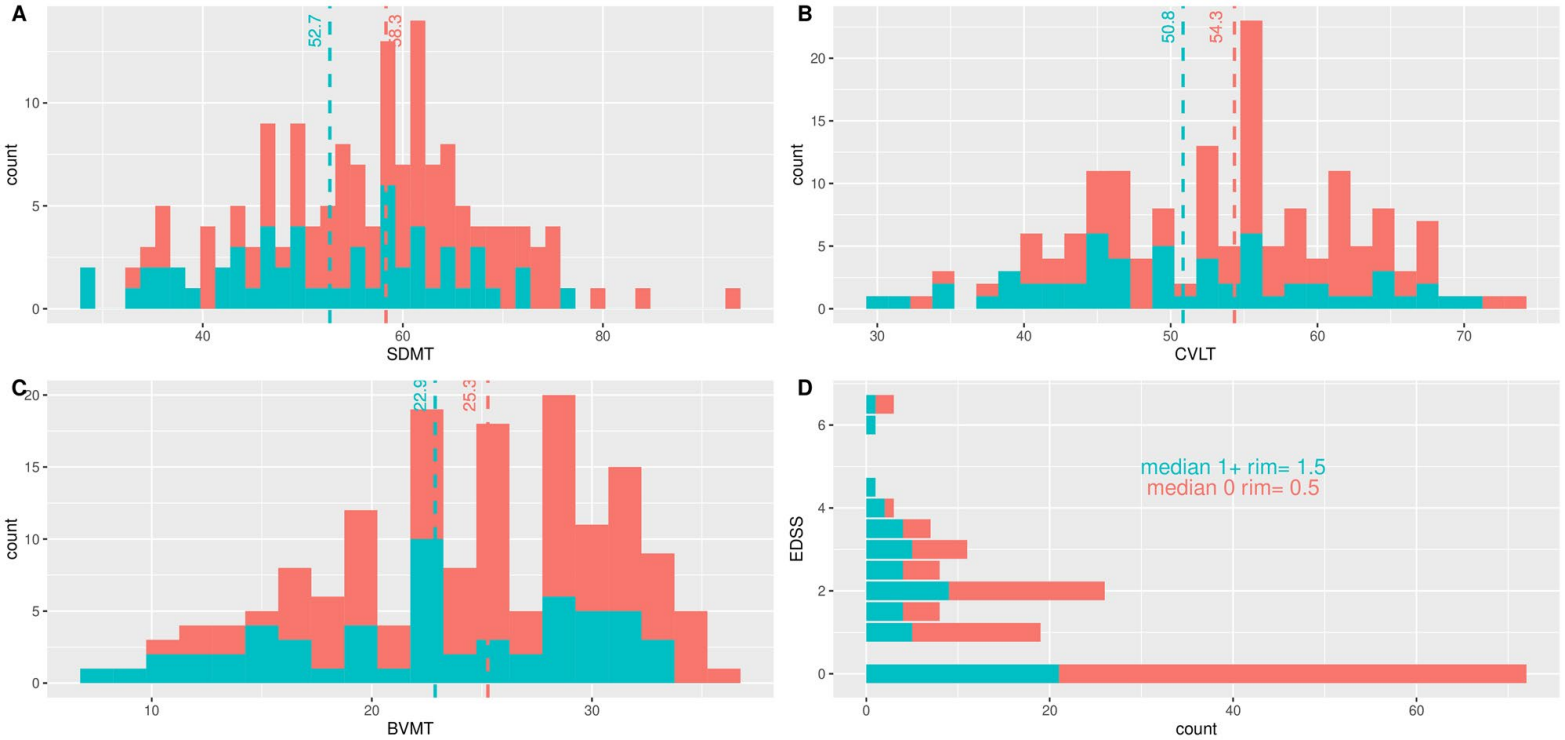
Distribution of disability scores comparing patients with zero (0: red) rim+ lesions versus patients with at least one rim+ lesion (1+: blue). Dash lines represent the mean for each group. Patients with at least one rim+ lesion performed worse on average SDMT (**A**), CVLT-II (**B**), BVMT-R (**C**) and EDSS (**D**).

Figure 2 compares the sample distributions of cognitive scores of patients with and without rim+ lesions. The distributions of SDMT, CVLT-II, and BVMT-R have a similar spread for both groups of patients; however, each sample mean is consistently higher for patients without rim+ lesions. Figure 2 also shows that more patients without rim+ lesions have higher SDMT, CVLT-II, and BVMT-R scores, as the right tails of the plots correspond to scores exclusively from patients without rim+ lesions. The distribution of EDSS also indicates that the majority of patients without rim+ lesions have lower EDSS scores. Given these results, we hypothesized that, on average, patients without rim+ lesions have lower disability mean scores than patients with at least one rim+ lesion. To test this hypothesis, we use a multivariate regression model approach and obtained the corresponding multivariate analysis of variance (MANOVA) and subsequent Analysis of Variance (ANOVA) tables for each clinical score.

**Figure 3:**
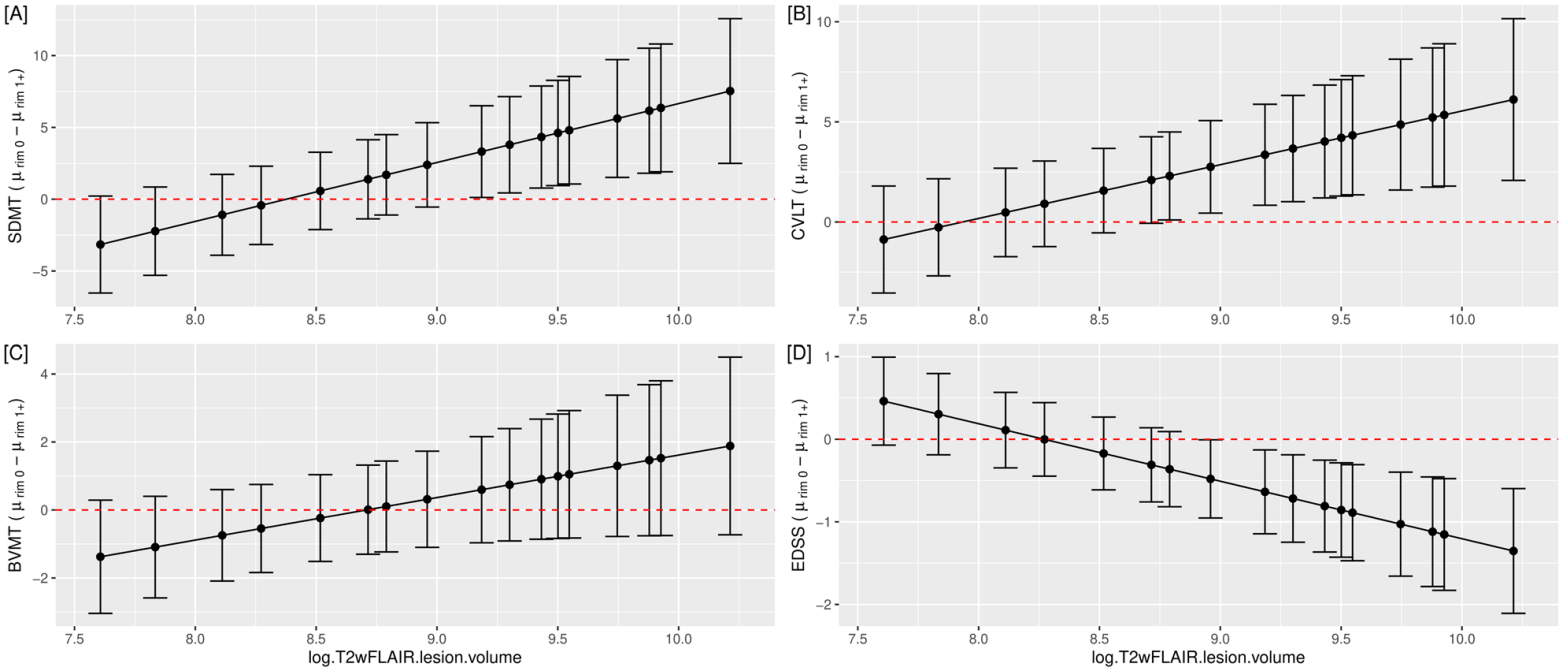
Association of rim+ lesions with disability measures after accounting for clinical and imaging covariates. Patients with both higher total lesion volume on FLAIR and at least one rim+ lesion performed worse on SDMT (**A**), CVLT-II (**B**), and EDSS (**D**) as demonstrated by 95% confidence intervals of the difference of mean disability scores between patients with zero rim+ lesions minus patients with at least one rim+ lesion. The 95% confidence intervals of the means are model-based estimates. The final model included current treatment duration, age, gender, total lesion volume on FLAIR, and the number of rim+ lesions (statistically significant covariates). Performance on BVMT-R (**C**) did not reach clinical significance in the model.

Table 2 summarizes the p-values obtained from the MANOVA and ANOVA tables. We aimed to investigate the effect of possible interactions between the number of rim+ lesions (0 versus at least 1) and other patient-level covariates on disability scores. The only statistically significant interaction was between rim+ lesions and total lesion volume on FLAIR imaging on disability outcome measures (p = 0.006), but each of the variables was also significant (number of rim+ lesions, p = 0.010 and log-TLV, p < 0.001). Other statistically significant patient-level covariates were current treatment duration, age, and gender with p-values of 0.012, < 0.001, and 0.038, respectively. Individual analysis of variance tables for each score confirmed that the interaction effect between log-TLV and number of rim+ lesions (0 versus at least 1) was statistically significant for SDMT ($\widehat{\beta }$ = − 4.11, p = 0.010), CVLT-II ($\widehat{\beta }$ = − 2.68, p = 0.030), and EDSS ($\widehat{\beta }$ = 0.70, p = 0.001). That is, as the overall total lesion burden increases, patients with at least one rim+ lesion on QSM have, on average, lower SDMT and CVLT-II mean scores. As TLV increases, EDSS mean scores for patients with at least one rim+ lesions are higher than for patients without rim+ lesions. There was no significant difference in performance found between groups for BVMT-R. Figure 3 displays a graphical representation of model-based cognitive and EDSS mean difference estimates between patients with zero rim+ lesions versus at least one rim+ lesion and their 95% confidence intervals. These results highlight the impact of increasing TLV and the presence of only one rim+ lesion.

**Table 2:** Summary of p-values from the Multivariate Analysis of Variance (MANOVA) table and subsequent Analysis of Variance (ANOVA) tables for each score. The final multivariate model for SDMT, CVLT-II, BVMT-R, and EDSS as vectors of response variables included current Treatment Duration, Age, Gender, No. RIM of + lesions, log T2wFLAIR lesion volume, and the interaction term No. RIM * logT2wFLAIR.lesion.volume as covariates. This report gives the p-values associated with the MANOVA Pillai test and the approximated F-statistics for each score (ANOVA). The bolded values represent the significant association of the interaction term (No. RIM * logT2wFLAIR.lesion.volume) with the clinical outcomes.

	MANOVA	ANOVA for each cognitive score			
		SDMT	CVLT-II	BVMT-R	EDSS
(Intercept)	< 0.001	< 0.001	< 0.001	< 0.001	0.386
Current treatment duration	0.012	0.389	0.137	0.513	0.009
Age	< 0.001	< 0.001	0.030	< 0.001	< 0.001
Gender	0.038	0.508	0.003	0.592	0.841
No. RIM (0 rim + versus 1 + rim + lesion)	0.010	0.011	0.050	0.157	0.001
logT2wFLAIR.lesion.volume	< 0.001	< 0.001	0.294	0.002	0.607
No. RIM * logT2wFLAIR.lesion.volume	0.006	**0.010**	**0.030**	0.165	**0.001**

The marginal standardized effect values were computed to compare the marginal effect of total lesion volume, the presence /absence of rim+ lesions, and their interaction on each clinical score (Appendix 4, online Supplementary File). Standardized effect values are directly comparable in magnitude and direction and can be used for ranking the effect sizes in terms of the number of standard deviations^44^. As an example, the marginal standardized effect of total lesion volume on SDMT is − 0.45, and the standardized effect of the presence/absence of rim+ lesions is − 0.22, whereas the standardized joint effect is − 0.53. Similarly, if we rank the marginal standardized effect of these variables on logEDSS: total lesion volume is 0.10, the presence/absence of rim+ lesions is 0.39, and their interaction is 0.51.

### Association of QSM rim lesions and gray matter damage

In this secondary analysis, we modeled the association between cortical thickness and the presence of a rim+ lesion ($\widehat{\upbeta }$ = − 0.002, p < 0.001, 95% CI (− 0.076, − 0.008)), while controlling for other covariates. The final model also included Age ($\widehat{\beta }$ = − 0.049, p = 0.001, 95% CI (− 0.004, − 0.001)); importantly, TLV was not found to be associated with cortical thickness (p > 0.10). In a separate model, lower thalamic volume was associated with older patients (p = 0.038) and larger total lesion burden (p < 0.001); however, the association did not remain significant with rim+ lesions (p > 0.10) after controlling for age ($\widehat{\beta }$ = − 0.00013, p = 0.004), gender ($\widehat{\beta }$ = − 0.00032, p = 0.0318), and TLV ($\widehat{\beta }$ = − 0.00026, p < 0.001).

## Discussion

In this study, we demonstrated a meaningful impact of having at least one QSM rim+ lesion on both the clinical and imaging outcomes. Our study not only supports the capacity of QSM as an imaging tool to detect lesions with a rim of iron-laden microglia and macrophages^15,20,21^ but also demonstrated that the magnitude of the effect of overall lesion volume on disability depends on whether or not rim+ lesions are present.

The identification of rim+ lesions using QSM is based upon the visualization of a hyperintense signal, reflective of increased susceptibility due to iron deposition, at the rim of a subset of chronic lesions^20,21,45^. Traditional GRE MRI imaging, including susceptibility-weighted imaging (SWI) and GRE phase imaging, cannot accurately localize or quantify the susceptibility of iron due to difficulty arising from blooming artifacts, which may alter specificity for identification of lesions with a paramagnetic rim^46^. QSM, on the other hand, processes GRE phase data^47^ and deconvolutes the magnetic field to uncover the magnetic properties of tissues, localize the tissue magnetic source, and quantify tissue susceptibility; as a result, QSM identifies lesions with true iron deposition^19,48^. Our study harnessed the capacity of QSM to successfully identify lesions with a rim of iron-laden microglia and macrophages and assess their relationship with disability in patients with multiple sclerosis.

A multivariate model was selected to assess the relationship between rim+ lesions and disability, given that this approach can account for the correlation between multivariate clinical scores for an individual patient’s covariates. We found a significant joint effect of TLV and the presence of rim+ lesions on clinical scores. This novel observation highlights the importance of appropriately considering the combined joint effect of chronic active lesions and total lesions on clinical outcomes. In this study, we demonstrated that in patients with at least one rim+ lesion, as the overall FLAIR lesion burden increases, both cognition and EDSS worsen, reflective of the linear relationship of the two imaging variables. Recently, the relationship between chronic active MS lesions and performance on EDSS and processing speed, as measured by the SDMT, was explored utilizing phase imaging^13^; however, this study has essential differences. Our work expands upon these findings by incorporating the relationship with overall lesion volume and including additional domains of cognition, such as verbal and visuospatial memory as measured by the CVLT-II and BVMT-R, respectively. Through the use of QSM, we were able to identify the impact of one or more chronic active MS lesions on disability, while in the study of Absinta et al., the impact of phase rim lesions on disability was associated with four or more phase rim lesions^13^. Technical differences between the two imaging methods should be considered^18,19^ and warrant a thorough evaluation to determine which imaging approach most accurately identifies this subset of chronic lesions. Importantly, taken together, these two large cohort studies demonstrate that the presence of rim+ lesions is associated with a more aggressive phenotype of the disease. Future studies should be dedicated to understanding why these lesions are found in only a subset of patients.

Only a minority of our patients would be classified as cognitively impaired, and given this, as well as the fact that age was already accounted for in statistical models, we chose to keep the raw cognitive scores for the analysis. Our findings suggest that the relationship between total lesion burden, chronic active lesions, and cognition may be especially relevant for early, pre-clinical cognitive changes in MS. Such a subtle, early decline in processing speed and memory is functionally impactful, particularly for young patients who are likely to have significant social and occupational responsibilities. Our results may be informative in identifying patients that might benefit the most from early cognitive interventions. Although no association was found regarding the relationship between rim+ lesions and BVMT-R, a decreased performance was found as TLV increased. Attributing cognitive dysfunction to white matter lesion characteristics, mainly lesion volume, has yielded variable results in past studies^49^, likely related to the dominant role of cortical pathology^50^ or the lack of consideration of the white matter lesion’s impact on the brain’s overall connectivity network. In fact, our recent study revealed that, while cortical/subcortical pathology was strongly predictive of cognitive outcomes, adding information about the topological impact of white matter lesions on the brain’s connectivity network may improve accuracy^51^. Therefore, even our positive results regarding SDMT and CVLT-II should be interpreted with caution. In addition, *BICAMS* is a limited battery of cognitive assessments and is not meant to be a comprehensive neuropsychological evaluation^31^.

In a secondary analysis, we explored the influence of having at least one rim+ lesion on measures of gray matter integrity, intending to demonstrate an association of chronic active MS lesions with global neurodegeneration. A lower thalamic volume has been previously associated with having higher numbers of chronic active lesions, as measured by phase imaging^13^; although patients with QSM rim lesions had a smaller thalamic volume in the current study, the difference failed to reach significance in the regression model. Our results identified a strong influence of total lesion burden on thalamic volume, suggesting that diffuse damage to white matter connections may have a stronger influence on thalamic damage^52^. Nevertheless, our study is unique in showing the strong association with cortical gray matter and suggests that the presence of chronic active lesions may represent those patients with a more aggressive phenotype. Larger studies with healthy control populations are required to fully explore the influence of rim+ lesions on measures of tissue loss.

Chronic active MS lesions have traditionally been associated with progressive disease, which is based upon post-mortem histopathological studies^3^. However, given the bias toward progressive patients within these studies, it is unclear how prevalent chronic active lesions are in earlier stages of the disease. Studies utilizing phase imaging have demonstrated that over 50% of patients have at least one phase rim+ lesion in relapsing disease and represent approximately one-third of the lesions^53^. In our study, rim+ lesions represent a minority of chronic lesions (3.8%), and only 35.9% of patients had a rim+ lesion; again, this discrepancy may relate to differences between phase and QSM. Although other studies have similarly found that QSM rim+ lesions represent a minority of white matter lesions (9–20%)^15,20,25^, the amount of rim+ lesions in this patient cohort is relatively low. Importantly, when focusing on RR patients only, Harrison et al. found that only 6% of lesions demonstrated a hyperintense rim on QSM, suggesting that studies of earlier MS may reflect a lower prevalence of QSM rim+ lesions. Thus, the variability rim+ lesion detection may be related to differences among the cohorts and/or a lack of a specific criterion for identifying a hyperintense rim. Similar to other paramagnetic rim studies, QSM rim+ lesions are characterized visually; thus, variability in this definition among groups of readers is likely a contributing factor. Reviewers for this study were clearly conservative in their definition but importantly, demonstrated a strong agreement.

There were limitations to this study. The clinical cohort consisted of RRMS patients with a relatively low disease duration and disease burden. To obtain a more comprehensive picture of the impact of QSM rim+ lesions on cognition and physical disability, it is possible that a more impaired population, such as one with a large representation of progressive patients, may be more informative. Furthermore, our study was observational and only association statements can be drawn. In addition, our current slice thickness of 3 mm could limit detection of small rim+ lesions and underestimate the true prevalence; however, our sequence provides high in-plane resolution for improved excellent axial visualization and can be acquired within 4 min, making this a clinically feasible sequence. Our analysis regarding cortical thickness and thalamic volume are secondary analyses and only suggests that the presence of rim+ lesions may have a negative impact on cortical thickness, a measure of neurodegeneration. We did not see a significant association with thalamic volume, another measure of neuronal loss in MS; however, there was a trend. We acknowledge that these results are less robust, as compared to the main result, and would require larger datasets with either longitudinal data (to assess atrophy rates) or a comparison to healthy controls to confirm a clear association. As we move forward, larger, multi-center studies should standardize the most accurate imaging approach and rim lesion definition. Regardless of our low rim+ lesion number, our results still indicated that the presence of these lesions was associated with lower scores on disability and more cortical gray matter loss.

In conclusion, our study demonstrates the significant association of the presence of chronic active lesions on both clinical and imaging features of disability and highlights the relevance of identifying these lesions at each stage of the disease. Furthermore, our work promotes the use of QSM to identify chronic active lesions and as a tool to understand their role in clinical progression. Future work will include longitudinal studies building upon our findings to establish the role of rim+ lesions as a prognostic biomarker in MS and to further explore the quantitative aspects of QSM to assess response to novel therapeutic approaches targeting the innate immune response in the CNS^54^.

## Supplementary Information

Supplementary Information.

## Abbreviations

MS: Multiple sclerosis
RR: Relapsing–remitting
BVMT-R: Brief Visuospatial Memory Test-Revised
CVLT-II: California Verbal Learning Test-II
EDSS: Expanded disability status score
FLAIR: Fluid-attenuated inversion recovery imaging
QSM: Quantitative susceptibility mapping
SDMT: Symbol Digit Modalities Test
