# Supplementary material for: Disease correlates of rim lesions on quantitative susceptibility mapping in multiple sclerosis
Source: Sci Rep. 2022 Mar 15;12:4411. doi: 10.1038/s41598-022-08477-6 (PMC8924224; doi:10.1038/s41598-022-08477-6)
Supplement: Supplementary file 1 — Supplementary Information. [file 41598_2022_8477_MOESM1_ESM.docx]

**Disease correlates of quantitative susceptibility mapping rim lesions in multiple sclerosis**

**Online supplemental file**

Melanie Marcille, BA^1^, Sandra Hurtado Rúa, PhD^2^, Charles Tyshkov, MD^3^, Abhishek Jaywant, PhD^4^, Joseph Comunale, MD^5^, Ulrike W. Kaunzner, MD, PhD^1^, Nancy Nealon, MD^1^, Jai S. Perumal, MD^1^, Lily Zexter, BA ^1^, Nicole Zinger, BS^1^, Olivia Bruvik ^1^, Yi Wang, PhD ^5^, Elizabeth Sweeney, PhD^7^, Amy Kuceyeski, PhD^5,6^, Thanh D. Nguyen, PhD ^5^ and

Susan A. Gauthier, DO, MPH ^1,5,6^

^1^ Department of Neurology, Weill Cornell Medicine, New York, NY, USA

^2^Department of Mathematics and Statistics, Cleveland State University, Cleveland, OH, USA

^3^Department of Pediatrics, Weill Cornell Medicine, New York, NY, USA

^4^Department of Psychiatry and Rehabilitation Medicine, Weill Cornell Medicine, New York, NY, USA

^5^Department of Radiology, Weil Cornell Medicine, New York, NY, USA

^6^Feil Family Brain and Mind Institute, Weill Cornell Medicine, New York, NY USA

^7^Department of Population Health Sciences, Weill Cornell Medicine, New York, NY, USA

Corresponding Author:

Susan Gauthier, DO, MPH

Judith Jaffe Multiple Sclerosis Center

1305 York Avenue

New York 10021, NY

Tel: (646) 962-9800

Fax: (646) 962-0390

E-Mail: [sag2015@med.cornell.edu](mailto:sag2015@med.cornell.edu)

Running title: Rim lesions associate with disability

**Appendix 1.** Study Flowchart.


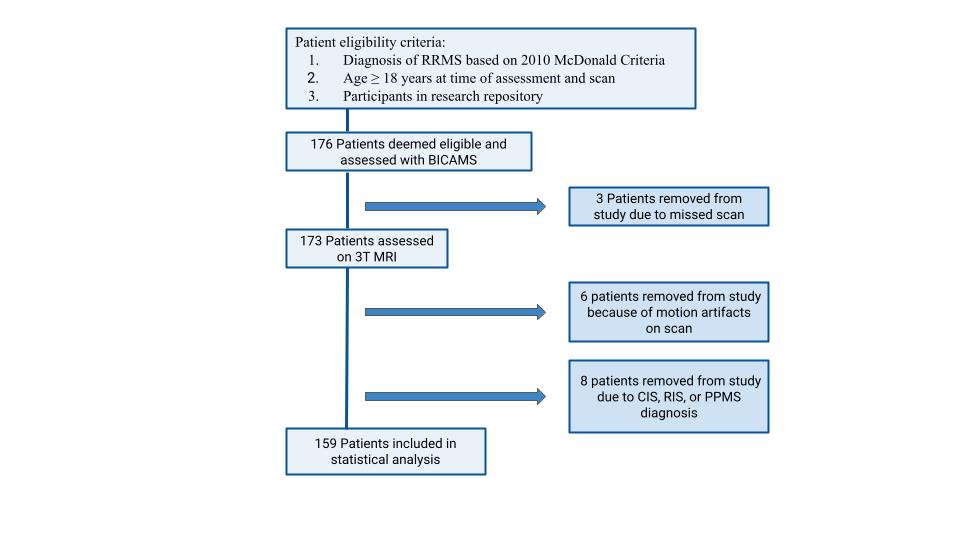


**Figure 1.** Study flowchart.

**Appendix 2. Multivariate regression model and MANOVA table**

Multivariate regression is a technique that models more than one response variable in a single regression model framework. Our cross-sectional retrospective study has four cognitive outcome variables per patient (SDMT, CVLT-II, BVMT-R, and EDSS) as well as several covariates of interest (current treatment duration, Age, Gender, No.rim.p, and logTLV.flair). Instead of ruing 4 independent univariate regression analyses, one for each outcome, we performed a multivariate analysis (see equation 1).

$$\left( \begin{matrix} {SDMT}_{\boldsymbol{i}} \\ {CVLT}_{\boldsymbol{i}} \\ \begin{matrix} {BVMT}_{\boldsymbol{i}} \\ \boldsymbol{log}\left( \boldsymbol{EDSS}_{\boldsymbol{i}}\boldsymbol{+0.1} \right) \end{matrix} \end{matrix} \right) \boldsymbol{=\beta}_{\boldsymbol{0}} {\boldsymbol{+}\boldsymbol{\beta}_{\boldsymbol{1}} \boldsymbol{No.rim}\boldsymbol{.p}}_{\boldsymbol{i}}\boldsymbol{+}\sum_{\boldsymbol{j=2}}^{\boldsymbol{6}} \boldsymbol{\beta}_{\boldsymbol{j}} \boldsymbol{x}_{\boldsymbol{ji}}\boldsymbol{+}\sum_{\boldsymbol{j=7}}^{\boldsymbol{10}} \boldsymbol{\beta}_{\boldsymbol{j}} \boldsymbol{x}_{\boldsymbol{ji}}\boldsymbol{No.rim.p}_{\boldsymbol{i}}\boldsymbol{+}\boldsymbol{\varepsilon}_{\boldsymbol{i}}\boldsymbol{,}$$

***(Eq. 1)***

Where each $\boldsymbol{\beta}_{\mathbf{j}}$**; j=0,1,2,…,10**  is a $1 \times4$vector of parameters quantifying the linear association between each cognitive score and covariates ; $\boldsymbol{\varepsilon}_{\mathbf{i}}$is the $1 \times4$ vector of error terms and it is assumed to be multivariate normal with a mean zero vector and a covariance-variance matrix given by $\Sigma$, thus the error terms associated with different outcomes may be correlated.

The left-hand-side of equation 1 denotes the vector of cognitive scores (multiple outcome variables) for patient $i$and the right-hand side shows the linear model with two-way interactions. Our primary goal is to estimate each vector of beta parameters ($\boldsymbol{\beta}_{\mathbf{j}}\mathbf{)}$ as they quantify the association between cognitive scores and the number of rim+ lesions per patient (No.rim.p: zero versus more than one rim+ lesions) while adjusting for other patient-level covariates such as logTLV.flair ($x_{2})$, sex ${(x}_{3})$, age ( $x_{4})$, disease duration ${(x}_{5})$and current treatment ($x_{6}$) as well as covariate interactions of the form $x_{\mathrm{ji}}{No.rim.p}_{i}$, j=2,…6).

The model described in Equation 1 is also known as a multivariate multivariable regression model [1]. The terms multivariate and multivariable are often used interchangeably in medical research. However, multivariate analysis refers to the joint analysis of multiple outcomes in one modeling framework whereas multivariable analysis deals with only one outcome and multiple covariates[2].

We aim to quantify the association between cognitive scores and the presence/absence of rim+ lesions while accounting for clinical patient-level covariates and overall lesion size. We did not include atrophy and cortical thinning measurements as we did not intend to model the marginal association (after accounting for atrophy and cortical thinning) between the number of rim+ lesions and cognitive scores.

We now discuss some of the advantages of multivariate regression models [3] over univariate regression models. First, a multivariate regression model allows us to quantify the relationship between all four disability scores as outcomes and all patient-level covariates in one framework. Multivariate regression controls over the family-wise error rate and gives a more realistic modeling framework than looking at a single variable, it also provides a more powerful test of significance compared to univariate techniques. Lastly, many statistical software, including R, estimate the parameters in equation 1 as well as perform hypothesis testing [4].

Multiple Analysis of Variance (MANOVA) table extends the Analyses of Variances (ANOVA) table to studies with two or more related outcomes/dependent variables while controlling for the correlations among them. The MANOVA table displays a p-value based on a multivariate test statistic. We reported a p-value based on an F approximation of the Pillai test statistics for each parameter. Although the MANOVA p-values provide a more powerful test of significance compared to univariate techniques, we also reported the ANOVA tables, which are based on t-statistics and look at each score independently.

**Appendix 3. Model Selection**

We selected the final multivariate multiple regression model via model selection using the AIC and BIC selection criteria. In particular, we implemented the stepwise backward selection procedure using [5] by specifying the full model with all possible two-way interactions(see equation 1) and the lower bound model as the model without interactions. All final models were selected using a stepwise backward procedure [5] with a 0.10 significance level.

Table 1 below displays the p-values from the Multivariate Analysis of Variance (MANOVA) table. The full multivariate model for SDMT, CVLT-II, BVMT-R, and EDSS as a vector of response variables included current Treatment Duration, Age, Gender, No. RIM of+ lesions, log T2wFLAIR lesion volume, and all two-way interaction terms between No. RIM + lesions and other covariates.

|  | **MANOVA**  **(p-values)** |
| --- | --- |
| **(Intercept)** | <0.001 |
| **Current Treatment Duration** | 0.026 |
| **Age** | <0.001 |
| **Gender** | 0.040 |
| **logT2wFLAIR.lesion.volume** | <0.001 |
| **No. RIM (0 rim+ versus 1+ rim+)** | 0.002 |
| **No. RIM * logT2wFLAIR.lesion.volume** | 0.013 |
| **No. RIM * Gender** | 0.115 |
| **No. RIM * Age** | 0.619 |
| **No. RIM * Current Treatment Duration** | 0.186 |

**Table 1.** Summary of p-values from the full Multivariate Analysis of Variance (MANOVA). The initial multivariate model for SDMT, CVLT-II, BVMT-R, and EDSS as a vector of response variables included the following covariates: Current Treatment Duration, Age, Gender, No. RIM of+ lesions, log T2wFLAIR lesion volume, and all of the interaction terms between No. RIM and other covariates. This report gives the p-values associated with the MANOVA Pillai test.

Table 2 summarizes the p-values obtained from the multivariate analysis of variance (MANOVA) table. The final model (MANOVA) included a statistically significant interaction effect of rim+ lesions and total lesion volume on FLAIR imaging on disability outcome measures (p=0.006). Furthermore, the number of rim+ lesions (0 versus at least 1) and log-TLV were also significant (p=0.010 and p<0.001, respectively). Other statistically significant patient-level covariates were treatment duration, age, and gender with p-values of 0.012, <0.001, and 0.038, respectively.

|  | **MANOVA**  **(p-values)** |
| --- | --- |
|  | **(p-values)** |
| **(Intercept)** | <0.001 |
| **Current Treatment Duration** | 0.012 |
| **Age** | <0.001 |
| **Gender** | 0.038 |
| **No. RIM (0 rim+ versus 1+ rim+)** | 0.010 |
| **logT2wFLAIR.lesion.volume** | <0.001 |
| **No. RIM * logT2wFLAIR.lesion.volume** | 0.006 |

**Table 2.** Summary of p-values from the Multivariate Analysis of Variance (MANOVA) table and subsequent Analysis of Variance (ANOVA) tables for each score. The final multivariate model for SDMT, CVLT-II, BVMT-R, and EDSS as a vector of response variables included current Treatment Duration, Age, Gender, No. RIM of+ lesions, log T2wFLAIR lesion volume, and the interaction term No. RIM * logT2wFLAIR.lesion.volume as covariates. This report gives the p-values associated with the MANOVA Pillai test and the approximated F-statistics for each score (ANOVA)

**Appendix 4. Marginal Effects**

| **[A]** 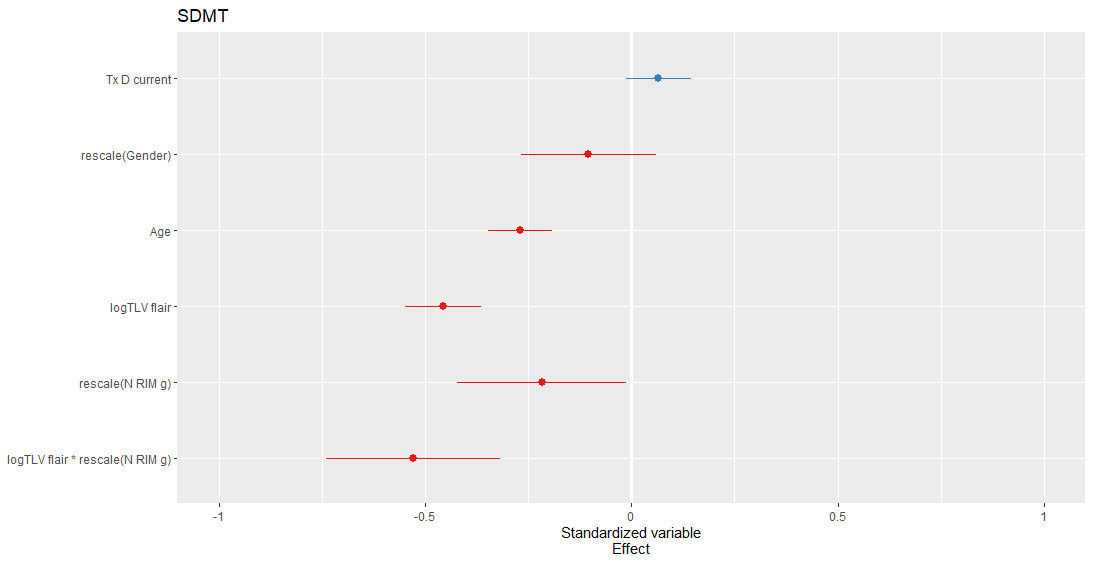 | **[B]** 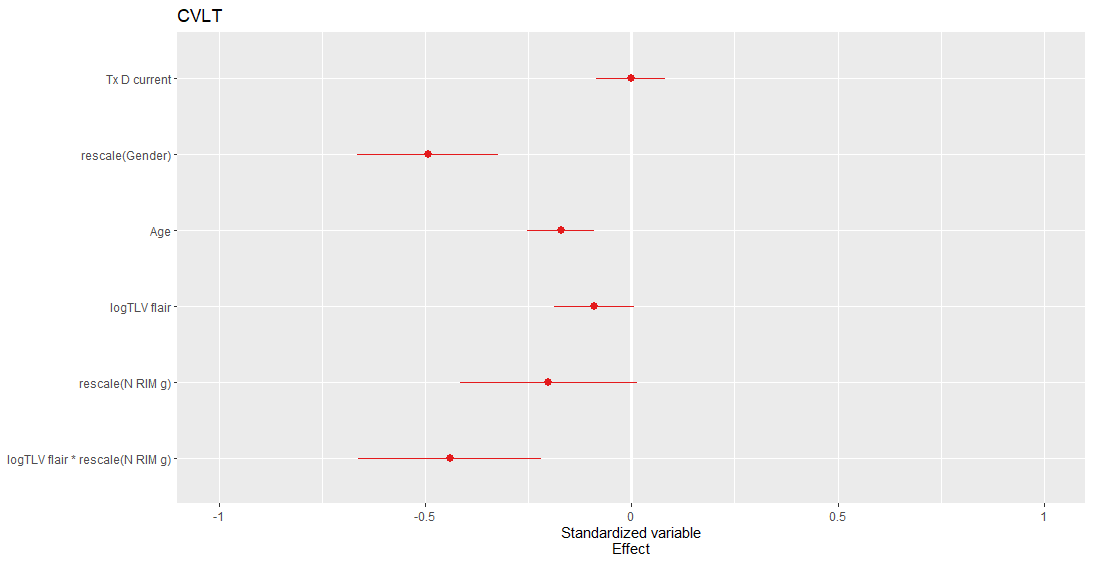 |
| --- | --- |
| **[C]** 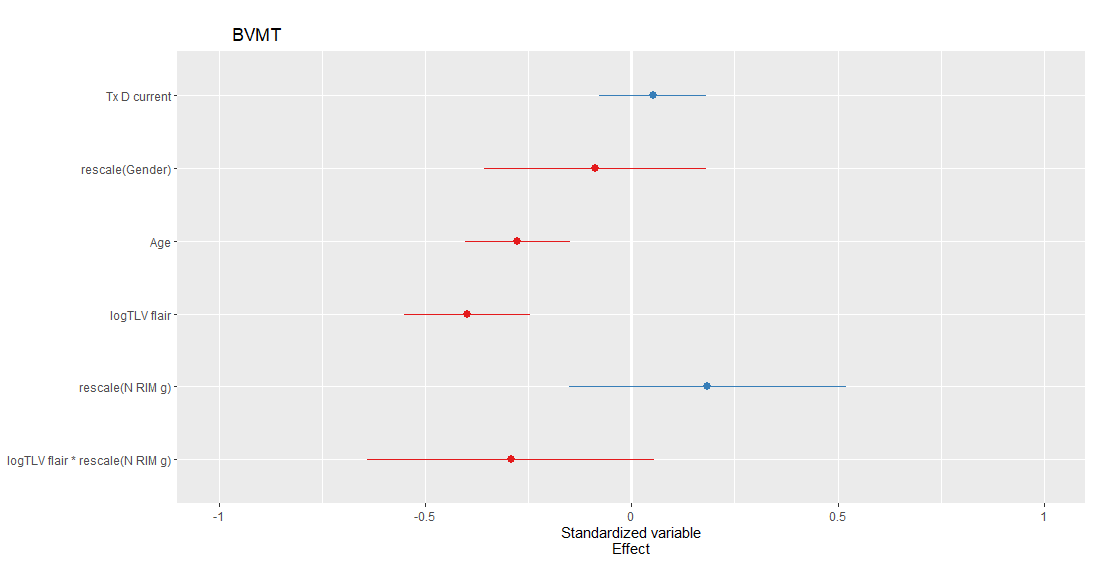 | **[D]** 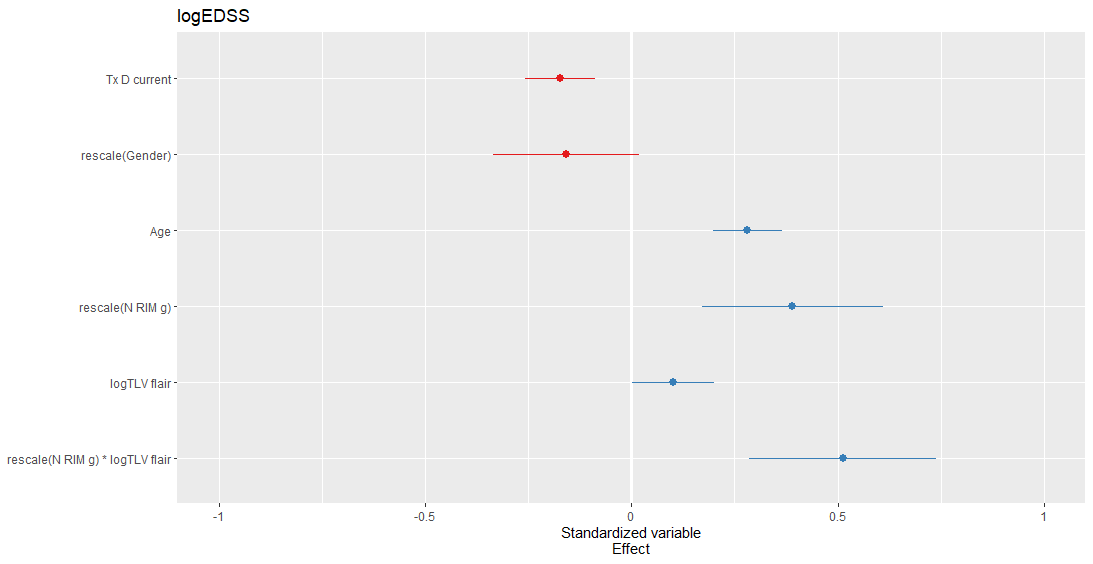 |

**Figure 2.** Plot of the standardized effect values [6]. The resulting values are then directly comparable in magnitude and direction and can be used for ranking the effect sizes in terms of the number of standard deviations.

**Appendix 5. Percentage of Model Variance Explained by RIM, Volume and their interaction using Multiple Regression Models**

|  | **(% of the Model Sum of Squares explained by each covariate.)** | | | |
| --- | --- | --- | --- | --- |
| **Covariate** | **SDMT** | **CVLT-II** | **BVMT-R** | **EDSS** |
| **No. RIM (0 rim+ versus 1+ rim+ lesion)** | 16.08 | **14.78** | 11.54 | 8.16 |
| **logT2wFLAIR.lesion.volume** | **34.50** | 12.35 | **36.96** | 11.83 |
| **No. RIM * logT2wFLAIR.lesion.volume** | 11.50 | 10.64 | 4.27 | **18.29** |

**Table 3.** Summary of the percentage (%) of model variance explained by each covariate based on subsequent Analysis of Variance (ANOVA) tables for each score). The percentage was computed with respect to the model sum of squares, not the total sum squares, and can be used to rank the variable contribution to the model sum of squares. The highest percentage is bolded.

**Appendix 6. Correlation analysis between Cognitive scores and volumetric measurements**


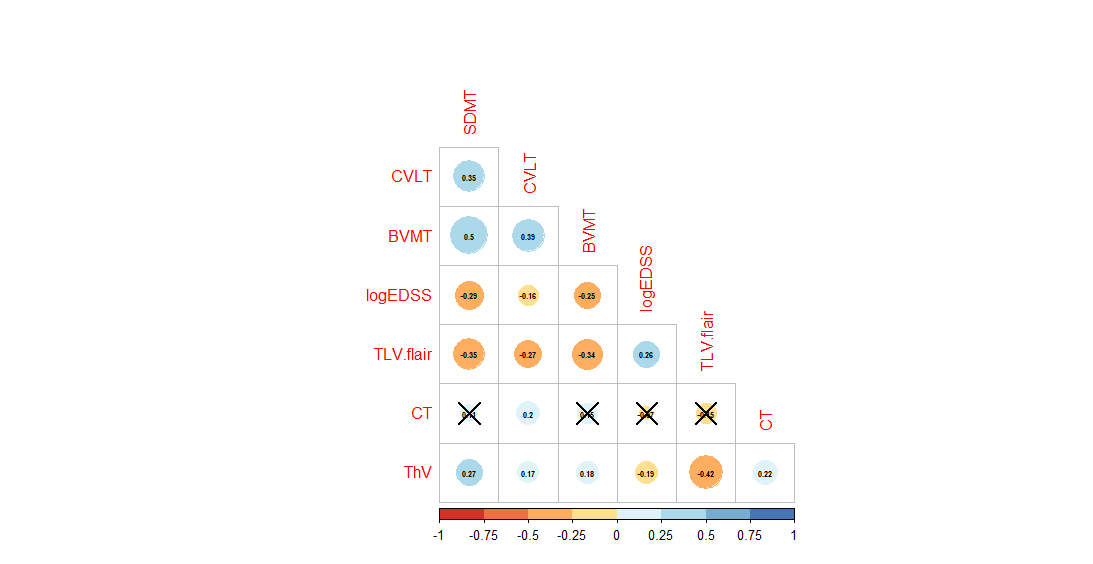


**Figure 3**. Plot of the correlation matrix of cognitive scores (SDMT, CVLT, BVMT, logEDDS) and volumetric measurements (TLV.frail, CT, ThV). The color and size of the circle represent the strength of the correlation. Printed values correspond to correlation coefficients while crossed values represent a non-statistically significant correlation (alpha=0.05)

**Appendix 7.** List of most relevant R packages used in this analysis

A list of the R packages used is below:

- {stats} : Multivariate model fitting
- {qtlmt}: Select a multivariate multiple regression model via model selection.
- {car}: ANOVA/MANOVA tables
- {effects}: Contrasts, effects
- {ggplot2, gridExtra, cowplot}: Plots

**References**

[1] Reboldi G, Angeli F, Verdecchia P: Multivariable Analysis in Cerebrovascular Research: Practical Notes for the Clinician. Cerebrovasc Dis 2013;35:187-193. doi: 10.1159/000345491

[2] Mohammad Ebrahimi Kalan, MS, Rime Jebai, MS, Elaheh Zarafshan, MS, Zoran Bursac, PhD, Distinction Between Two Statistical Terms: Multivariable and Multivariate Logistic Regression, Nicotine & Tobacco Research, Volume 23, Issue 8, August 2021, Pages 1446–1447, https://doi.org/10.1093/ntr/ntaa055

[3] Johnson RA, Wichern DW. Applied Multivariate Statistical Analysis, Upper Saddle River, NJ: Pearson Prentice-Hall.; 2007.

[4] John Fox and Sanford Weisberg (2019). An {R} Companion to Applied Regression, Third Edition. Thousand Oaks CA: Sage. URL: https://socialsciences.mcmaster.ca/jfox/Books/Companion/

[5] Riyan Cheng (2017). qtlmt: Tools for Mapping Multiple Complex Traits. R package version 0.1-6. <https://CRAN.R-project.org/package=qtlmt>

[6] Andrew Gelman. (2008). “Scaling regression inputs by dividing by two standard deviations.” *Statistics in Medicine* 27: 2865--2873.
